# Inconsistencies Exist in National Estimates of Eye Care Services Utilization in the United States

**DOI:** 10.1155/2015/435606

**Published:** 2015-08-09

**Authors:** Fernando A. Wilson, Jim P. Stimpson, Yang Wang

**Affiliations:** ^1^College of Public Health, University of Nebraska Medical Center, 984350 Nebraska Medical Center, Omaha, NE 68198-4350, USA; ^2^School of Public Health, City University of New York, 55 W. 125th Street, New York, NY 10027, USA

## Abstract

*Background*. There are limited research and substantial uncertainty about the level of eye care utilization in the United States. *Objectives*. Our study estimated eye care utilization using, to our knowledge, every known nationally representative, publicly available database with information on office-based optometry or ophthalmology services. *Research Design*. We analyzed the following national databases to estimate eye care utilization: the Medical Expenditure Panel Survey (MEPS), National Health Interview Survey (NHIS), Joint Canada/US Survey of Health (JCUSH), Behavioral Risk Factor Surveillance System (BRFSS), and the National Ambulatory Medical Care Survey (NAMCS). *Subjects*. US adults aged 18 and older. *Measures*. Self-reported utilization of eye care services. *Results*. The weighted number of adults seeing or talking with any eye doctor ranges from 87.9 million to 99.5 million, and the number of visits annually ranges from 72.9 million to 142.6 million. There were an estimated 17.2 million optometry visits and 55.8 million ophthalmology visits. *Conclusions*. The definitions and estimates of eye care services vary widely across national databases, leading to substantial differences in national estimates of eye care utilization.

## 1. Introduction

A recent report by the American Optometry Association (AOA) suggests that optometrists (OD) perform 88 million comprehensive eye exams annually, comprising 85% of all eye exams, compared to only 16 million (15%) exams performed by ophthalmologists (MD) [1]. These statistics have been widely cited in various reports on the eye care industry, and they have been used to demonstrate that most Americans rely on optometrists for their eye care needs [1–3]. However, the methodology used to generate these estimates is not available in the report, and there is significant uncertainty concerning the true number of eye care services that are being delivered in the US by optometrists, ophthalmologists, or other eye care professionals. To our knowledge, prior research examining the consistency and validity of current national estimates of eye care utilization across major publicly available healthcare databases does not exist. The uncertainty over utilization relative to the supply of eye care professionals—and thus uncertainty over whether the eye care needs of Americans are being met—has important implications for the creation and funding of programs and policies that impact either utilization or supply in the eye care market. Our study estimates eye care utilization using every known nationally representative, publicly available database with information on office-based optometry or ophthalmology services.

## 2. Methods

To examine this issue, we analyzed the following publicly available databases: the Medical Expenditure Panel Survey (MEPS), National Health Interview Survey (NHIS), Joint Canada/US Survey of Health (JCUSH), and the National Ambulatory Medical Care Survey (NAMCS). To our knowledge, these databases contain all nationally representative data on office-based eye care utilization that are available to the public in the US. The NHIS is a national database collecting self-reported data on health and healthcare utilization and other measures using in-person interviews and is managed by the Centers for Disease Control and Prevention (CDC) [4]. The MEPS uses a subsample of NHIS respondents to provide detailed healthcare and other pieces of information for individuals and also includes a survey of respondents' medical providers [5]. The Agency for Healthcare Research and Quality (AHRQ) is responsible for administering the MEPS database. The JCUSH database is conducted by the CDC and Statistics Canada and consisted of random telephone surveys between November 2002 and March 2003 in both US and Canada [6]. JCUSH data are nationally representative for the US, providing information on eye care utilization. Finally, the NAMCS is a national database of physicians conducted by the CDC and provides clinical data on visits to ophthalmologists in the US [7].

In addition to the above databases, we also examined the Behavioral Risk Factor Surveillance System (BRFSS), which provides eye care utilization data restricted to adults aged 40 and over in a limited number of states [8]. We estimated the annual total number and percent of adults aged 18 years and older (40 years and older for BRFSS) who utilized eye care services and also the total number of eye care visits for databases providing these data. All analyses were weighted and adjusted for complex survey design using STATA 13 (College Station, TX).

## 3. Results

Table 1 shows a wide range of definitions and estimates of eye care services. MEPS differentiates between optometrists and ophthalmologists. The NHIS and JCUSH do not differentiate eye care professionals or their practice settings, and NAMCS only provides data on ophthalmologists. The weighted number of adults seeing or talking with any eye doctor ranges from 87.9 million for NHIS to 99.5 million for JCUSH. Total number of visits annually is 142.6 million from JCUSH. By comparison, MEPS results suggest that there are about 45 million adults visiting office-based optometrists and ophthalmologists annually, totaling 72.9 million visits. MEPS is the only database providing information on optometry specifically, and it indicates there are 14.6 million people utilizing optometry services annually in the US, resulting in 17.2 million visits. MEPS and NAMCS provide data on ophthalmology-only visits; MEPS estimates 55.8 million ophthalmology visits in 2012 compared to 50.3 million for NAMCS in 2010. NHIS indicated there were 89.3 million dilated eye exams performed in 2008—the most recent year available for this measure.

Table 2 provides BRFSS weighted estimates of eye exams performed annually. Unfortunately, BRFSS collected eye care data for only 11 states in the period 2008–10 and only for persons aged 40 and older. 62.4% of respondents in these states had their eyes examined by a doctor or eye care professional. This compares to a range of 45–55% for persons aged 40 and over in the NHIS and JCUSH databases (results not shown). The weighted percentage of respondents reporting a dilated eye exam (49.5%) is similar to the results reported in the 2008 NHIS (47.9%; not shown).

In contrast to the above analyses, Table 3 provides estimates reported by AOA Excel and Jobson Medical Information in “The State of the Optometric Profession: 2013.” This report states that optometrists perform 88 million refractive eye exams annually. This statistic has been widely cited in various reports on the eye care industry. Unfortunately, we were not able to replicate or find information on the origin of this figure. It seems to be based on an analysis by Jobson's Practice Advancement Associates, but we could not find details of this analysis or the source of data used. However, this estimate is high compared to results from the other databases on eye care utilization. For example, the NHIS—used in several studies on eye care—shows that there are about 88 million adults who “see or talk to” any eye doctor (optometrists, ophthalmologists, or other professionals) for any reason in a year.

Conversely, the AOA Excel/Jobson report's finding that there are only 16 million exams performed by ophthalmologists in 2012 seems low. The NAMCS—a national survey of medical providers—shows that there were 50.3 million ophthalmology visits annually among adults in 2010 (and 55.5 million visits among all age groups (not shown in Table 2)). Using ICD-9-CM procedure codes for eye examination (95.00–95.09), NAMCS data show that ophthalmologists performed 22.2 million exams for all age groups in 2010 (not shown in tables). 2010 was the most recent survey year for NAMCS and there were substantial missing values for the procedure codes, so the actual number of eye exams performed by ophthalmologists may be significantly higher today. 2012 MEPS data for ophthalmologists show that 31.5 million adults utilize ophthalmologists at least once in a year resulting in 55.8 million total visits, compared to 50.3 million visits from NAMCS.

The wide range of eye care utilization estimates is likely a function of the variance in question wording (see footnotes of Table 1), but it is also possible that there are differences in survey design and populations studied across the databases. Therefore, we compared utilization of office-based physicians and receipt of routine medical checkups for MEPS, NHIS, BRFSS, and NAMCS (Table 4). The difference between the lowest and highest number of people receiving medical checkups varied by about 4% across the databases. Similarly, there was a 1.5% difference in weighted number of visits between MEPS and NAMCS. These results suggest that variations in measurement of eye care service utilization are unlikely due to differences in survey design and instead are related to differences in measurement.

## 4. Discussion

We found that definitions and estimates of eye care services vary widely across databases, leading to substantial differences in national estimates of eye care utilization. Furthermore, only MEPS and the AOA report provide estimates on optometry services. However, the AOA estimate of optometry services may be high, and its estimate of ophthalmology services low, compared to other available databases. The methodology used to estimate these services was not provided and, thus, we cannot gauge the accuracy or limitations of their estimates. Interestingly, differences in national estimates of general physician-based medical care are relatively small across the databases.

Our study findings on the large differences in eye care utilization have wider implications for determining whether eye care professionals are currently meeting primary care needs in the US population or if there is an oversupply of professionals. One study suggests that one out of two adults aged 20 and over has eye sight problems—accounting for about 112.7 million Americans [8]. Our results suggest that the needs of persons with eye sight problems may be sufficiently met according to one survey (JCUSH) or that there may be underutilization of eye care services based on other surveys (MEPS/NAMCS). These estimates suggest that aggregate annual visits may range from 67.5 to 142.6 million in the US. The uncertainty over utilization relative to the supply of eye care professionals impacts decisions about the creation and funding of programs and policies that impact either utilization or supply of eye care services. It is likely that there will be increasing need for eye care services with the aging population in the US, but the empirical evidence is equivocal as to whether there is sufficient access to optometrists, ophthalmologists, and other professionals to meet the demand for services. The uncertainty highlighted in our study suggests a need for improved data collection efforts in eye care services, particularly the measurement of providers and services provided. An important priority of Health People 2020 is to decrease visual impairment in the US, and to help achieve this goal, an expert panel convened by the Centers for Disease Control and Prevention called for the establishment of a vision surveillance system [9–11]. Furthermore, the CDC recently issued a Funding Opportunity Announcement with the purpose of implementing a national vision surveillance system [12]. Existing data sources such as NHIS and BRFSS would be important components of this system, and the success of the surveillance system would require an effective assessment of eye care utilization [10–12]. However, our study emphasizes the importance of resolving uncertainties in estimates of eye care utilization across national surveys, thus providing a solid foundation for a vision surveillance system. Our research may also signal a need to examine inconsistencies in national estimates for other healthcare professions, particularly auxiliary or specialty services.

## Figures and Tables

**Table 1:** Weighted number and percentage of adults aged 18 and older utilizing eye care services and weighted number of eye care visits in the United States stratified by nationally representative database.

	Weighted number of adults	Weighted number of visits	Weighted percent of adults visiting an eye care professional
Seeing or talking with any eye doctor in any setting in 12 months:			
2012 National Health Interview Survey (NHIS)^1^	87,850,196	N/A	38.0
2004 Joint Canada/US Survey of Health (JCUSH)^2^	99,472,902	142,634,037	48.3

Number of adults visiting an office-based optometrist in 12 months (ophthalmologists are excluded):			
2012 MEPS^3^	14,556,138	17,183,610	6.1

Number of adults visiting an office-based ophthalmologist in 12 months (optometrists are excluded):			
2012 MEPS^4^	30,434,241	55,756,866	12.8
2010 National Ambulatory Medical Care Survey (NAMCS)^5^	N/A	50,346,592	N/A

Having a dilated eye exam in 12 months:			
2008 NHIS^6^	89,335,468	N/A	40.4

^1^NHIS respondents were asked, “During the past 12 months, have you seen or talked to any of the following health care providers about your own health? … An optometrist, ophthalmologist, or eye doctor (someone who prescribes eyeglasses).” Possible responses included “Yes,” “No,” “Refused,” and “Don't know.” This question was only asked to adults aged 18 and over.

^2^2004 is the most recent survey year. JCUSH surveyed adults aged 18 and over by telephone. JCUSH respondents were asked, “In the past 12 months, how many times have you seen or talked with the following health care providers about your own health? … An eye doctor including other people that prescribe lenses (such as an ophthalmologist or optometrist)?” Possible responses included a numerical response, “Refused,” and “Don't know.” Estimates in Table 2 were restricted to US residents only.

^3^MEPS respondents were asked about any medical events and corresponding medical care visits occurring in the prior 12 months. For each office-based medical care visit, respondents were also asked “What type of medical person did you talk to on {Visit Date}?” Possible responses included “optometrist” in addition to other specialties. In addition, MEPS supplements self-reported data on medical care utilization with surveys of respondents' medical providers (MD or DO). The Medical Provider component of MEPS provides detailed information on ophthalmology services.

^4^MEPS supplements self-reported data on medical care utilization with surveys of respondents' medical providers. The Medical Provider component of MEPS provides detailed information on ophthalmology services.

^5^2010 is the most recent survey year. NAMCS is a national survey of office-based physicians who are nonfederally employed and engaged primarily in patient care activities.

^6^Respondents were asked, “When was the last time you had an eye exam in which the pupils were dilated? This would have made you temporarily sensitive to bright light.” Possible responses included “Less than one month”, “1–12 months”, “13–24 months”, “More than 2 years”, “Never”, “Refused”, “Don't know”.

**Table 2:** Weighted number and percentage of adults aged 40 and over utilizing eye care services in 11 states in 2008–2010, BRFSS^1^.

	Weighted number	Weighted percent
Eyes examined by any doctor or eye care provider in 12 months^2^	10,703,480	62.4
Having a dilated eye exam in 12 months^3^	7,126,244	49.5

^1^States included Alabama, Indiana, Iowa, Connecticut, Missouri, New Mexico, North Carolina, Tennessee, Wyoming, Arkansas, and Georgia. Only the most recent data were used for each state within the study period 2008–10.

^2^Respondents were asked, “When was the last time you had your eyes examined by any doctor or eye care provider?” Possible responses included “Within the past month,” “Within the past year,” “Within the past 2 years,” “2 or more years ago,” “Never,” “Refused,” and “Don't know/Not sure.”

^3^Respondents were asked, “When was the last time you had an eye exam in which the pupils were dilated? This would have made you temporarily sensitive to bright light.” Possible responses included “Within the past month,” “Within the past year,” “Within the past 2 years,” “2 or more years ago,” “Never,” “Refused,” and “Don't know/Not sure.”

**Table 3:** Total number of refractive eye exams performed annually in the US reported by AOA Excel and Jobson Medical Information LLC, 2012^1^.

	Number
Number of refractive eye exams performed by optometrists only	88 million
Number of refractive eye exams performed by ophthalmologists only	16 million
Total annual exams	104 million

^1^AOA Excel and Jobson Medical Information LLC. The State of the Optometric Profession: 2013 (available at http://www.reviewob.com/Data/Sites/1/soop_070120134.pdf. Accessed February 19, 2015).

**Table 4:** A comparison of utilization of office-based physician services across databases in the US.

	Weighted number of adults	Weighted number of visits	Weighted percent of adults visiting a physician
Number of visits to an MD/DO in 12 months by adults (18 years or older):			
2012 MEPS	160,637,294	829,664,093	67.7
2010 NAMCS	N/A	817,302,463	N/A

Number of adults (18 years or older) who received a routine checkup by physician in 12 months:			
2012 NHIS	154,957,097	N/A	67.1
2012 BRFSS	161,513,385	N/A	67.7
2012 MEPS	154,480,018	N/A	67.5
